# Progress in Medicine

**Published:** 1898-06-25

**Authors:** 


### PROGRESS IN MEDICINE.

### DISEASES OF THE RESPIRATORY ORGANS.

(*Continued from page* 203.)

**Bronchitis.**—Graudy[14] confirms Neelsen's statement that the casts in fibrinous bronchitis are composed of mucus, but found none of Curschman's spirals in them. Though apparently made up of fibres, with leucocytes and epithelial cells, there was no colour given by Weigert's fibrin stain. The epithelium of the bronchi was generally intact, the goblet cells very numerous, and the glands showed mucoid degeneration. Thus the origin of the casts was obvious at least in this instance, and the process analogous to membranous colitis. A. Davis[15] advocates sprays of ipecacuanha wine in the treatment of bronchitis. The patient should inhale during its use, and should spit out any of the fluid left in the mouth. Care should be taken to avoid vomiting and dyspnœa by any excess. A one per cent. solution of iodide of potassium may be given in the same way. The liquid extract of Hydrastis Canadensis is given by Sanger in chronic bronchitis with good results. Twenty to thirty drops are administered four times a day, but it is useless in the acute disease. A. Robin,[16] of Paris, has made out a strong case in favour of bleeding, not only in bronchitis but also in various forms of heart and kidney disease, in which he shows great results. Finny[17] notices a curious instance, viz., pneumothorax in a healthy man, without cough or pulmonary disease, which came on quite suddenly on two occasions. There was no temperature or quick pulse, but the left pleura was distended with air, the heart pushed over to the right side, and no effusion took place into the pleura. Complete recovery followed rest in bed. Pulmonary gangrene due to perforation of the œsophagus is rare, and almost always fatal, but Schroeder[18] has observed recovery after perforation by a bone which passed down the alimentary canal. A localised suppuration seems to have been set up in the mediastinum, and gangrene extended to the lung. The fœtid expectoration contained fragments of lung tissue, but the suppuration did not reach the pleura, and the patient was discharged well in nine weeks. Le Damany[19] has carefully investigated the nature and cause of sero-fibrinous pleurisies, and lays down that no other organisms, except B. tuberculosis, which may be present, are to be looked on as the cause. When they are primary they are tuberculous, whatever other microbes may be present; when secondary to pulmonary or other lesions, they may be non-tuberculous. It is only tuberculous pleurisies, therefore, which are due to microbial infection. Pneumococcal ones are nearly always purulent.

**Litten's sign** of the position of the lower border of the lung consists in an undulatory movement or shadow, which begins on both sides about the sixth space, and descends with inspiration as a distinct furrow for several spaces, reaching sometimes to the costal margin and returning with expiration. The recumbent position shows it best, and full daylight is desirable. Norman Gwyn[20] notices its value in marking the lower border of the lung, and its absence in extensive effusion, pleuritic adhesions, and emphysema. Litten states that it was present in every normal thorax out of many thousand observations, and, while in subphrenic abscess it commences high up, in presence of small effusions the line of movement is depressed.

C. Y. Biss,[21] in speaking of hæmoptysis, divides it into (1) that caused by phthisis through congestion, erosion of vessels, oozing from cavities, and the formation of minute aneurisms; (2) that from cardiac lesions through hyperæmia from mitral stenosis and incompetence, or through the formation of thrombi which become lodged in the lungs; (3) that caused by certain blood states, such as purpura and unknown lung conditions. Here he includes hæmorrhages, occasionally seen in people of all ages who have not been tubercular, and do not become so later on. The only exciting cause he refers to is some mechanical strain acting upon a naturally delicate lung. Besides the ordinary methods of treating hæmoptysis, such as rest, purgation, and turpentine, he aims at restricting the amount of fluid ingested, and gives a light solid diet with as little drink as possible. Hence, too, he refuses to give ice for the patient to suck, though he permits its outward application. Iodide of potassium is also recommended on theoretical grounds, but nothing is said of the utility of chloride of calcium.

[14] Brit. Med. Jour., Feb. 5. [15] Practit., Feb. [16] Inter. Med. Mag., Feb. [17] Brit. Med. Jour., April 9. [18] Brit. Med. Jour., April 23. [19] Amer. Jour. Med. Sci., March. [20] Johns Hopkins Bulletin. [21] Clin. Jour., Jan. 5.

